# Enhancing adsorption of U(VI) onto EDTA modified L. cylindrica using epichlorohydrin and ethylenediamine as a bridge

**DOI:** 10.1038/srep44156

**Published:** 2017-03-08

**Authors:** Shouzheng Su, Qi Liu, Jingyuan Liu, Hongsen Zhang, Rumin Li, Xiaoyan Jing, Jun Wang

**Affiliations:** 1 Key Laboratory of Superlight Material and Surface Technology, Ministry of Education, Harbin Engineering University, 150001, P. R. China; 2 Institute of Advanced Marine Materials, Harbin Engineering University, 150001, P. R. China; 3 Harbin Shipbuilding Engineering Design & Research Academy, Harbin, China; 4 Modern Analysis, Test and Research Center, Heilongjiang University of Science and Technology, Harbin 150027, P. R. China

## Abstract

Benefiting from strong coordination ability and unique vascular structure, EDTA modified L. cylindrica opens up an alternative way for uranium recovery from seawater. However, limitations, such as poor adsorption capacity and slow adsorption rate due to low graft ratio of EDTA via one-step esterification block its practical application. Here, a strategy for increasing the graft ratio is proposed in order to improve the adsorption performance. The strategy initially involves immobilization of epichlorohydrin (EPI) onto L. cylindrica and then ethylenediamine (EDA) is introduced via facile ring-opening reaction. EPI and EDA serve as a bridge between L. cylindrica and EDTA. The graft ratio is promoted (15.01 to 21.44%) contributing to the smaller steric hindrance of EPI and EDA than EDTA and improvement in adsorption performance. In addition, the adsorbent prepared by the new strategy exhibits excellent adsorption properties in simulated seawater.

Recovery of uranium from seawater has attracted great interest as a hot research topic recently, because of the heavy demand for uranium used in nuclear reactors^1^,^2^,^3^,^4^. In seawater, the total amount of uranium is about 4.5 billion tons, one thousand times more than the amount found in mineral ores on land^5^,^6^. However, its concentration is extremely low (~3.3 μg/L) while other metal ions in seawater have relatively high concentrations^7^,^8^. Therefore, the development of technologies and materials for realizing the selective extraction of uranyl ion from seawater is necessary and urgent. Many methods have been tried, including adsorption^9^,^10^,^11^, ion exchange^12^,^13^,^14^, chemical reduction^15^,^16^, biological processes^17^,^18^ and membrane processes^19^,^20^,^21^. Of these methods, adsorption is often preferred for its easy operation and lower cos; various kinds of organic adsorbents (such as such as amidoxime^22^,^23^,^24^, amine^25^,^26^,^27^, carboxylates^28^,^29^,^30^ functionalized adsorbents^31^) and inorganic adsorbents (such as mesoporous Mg(OH)_2_^32^,^33^,^34^, zero-valent iron^35^,^36^ and other functionalized inorganic adsorbents^37^,^38^,^39^,^40^,^41^,^42^) have been developed. However, limitations such as aggregation, complex post-treatment and nonbiodegradability precluded their practical applications in seawater.

Modified L. cylindrica has been used as an efficient adsorbent for the removal of heavy metal from aqueous solutions^43^,^44^,^45^. L. cylindrica is a natural porous fibrous vascular system. The fibers are disposed in a multidirectional array forming a mat-like structure. Because of its unique structure, L. cylindrica can deal with seawater efficiently and it is easy to implement. Moreover, due to abundant hydrophilic hydroxyl groups on the surface of fibers, selective functional groups are readily introduced to enhance the affinity and adsorption capacity for uranium^46^. However, to the best of our knowledge, the use of L. cylindrica for the recovery of uranium from seawater has not been investigated.

EDTA is a chelating agent which is widely used to sequester metal ions owing to its role as a hexadentate ligand (two amines and four carboxylate groups)^47^. It is well known that EDTA could form stable (1:1) complexes with rare earth ions in aqueous solution^48^. Inspired by the above, a facile route was developed for introducing EDTA onto the surface of L. cylindrica via esterification as in previous works^49^,^50^,^51^. However, the products prepared via the one-step method have a low graft ratio and poor adsorption. We reasoned that a high steric hindrance of EDTA caused a low graft ratio to be unfavorable for adsorption. To resolve this problem, a new strategy was proposed in which EPI and EDA were introduced to minimize space steric hindrance and improve graft ratio, which served as a bridge between L. cylindrica and EDTA. The effect of graft ratio of EDTA on the adsorption of U(VI) was discussed by comparing two routes. Adsorption kinetics and various isotherm models were evaluated to better understand the adsorption process. Finally, the adsorbent was employed to adsorb of U (VI) from simulated seawater so as to explore its potential application under real seawater.

## Result and Discussion

### Characterizations

**Figure 1:**
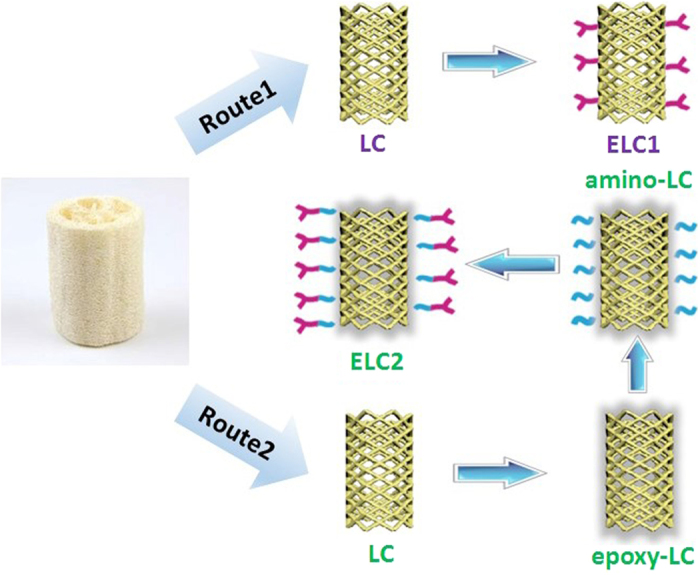
Steps involved in the preparation of EDTA modified LC by two routes.

The synthetic routes are illustrated in Fig. 1. In route1, ELC1 was prepared by reaction of EDTA dianhydride with LC directly. In the second route, EDTA was introduced onto L. cylindrica fibers by a series of chemical modifications, as listed below: (1) introduction of epoxy groups via reaction with L. cylindrica and EPI; (2) aminolyzation via a ring-opening reaction between the epoxy group and EDA; (3) immobilization of EDTA via facial acid-base reaction. EPI and EDA served as a bridge between L. cylindrica and EDTA.

**Figure 2:**
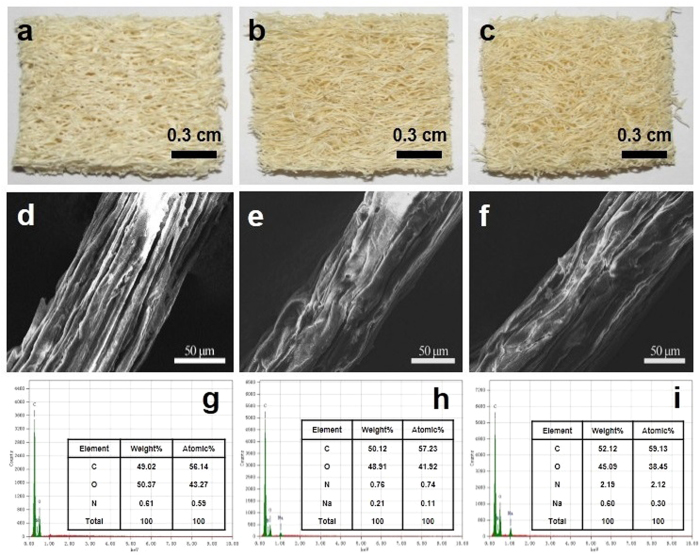
Digital photos of LC (**a**), ELC1 (**b**) and ELC2 (**c**); SEM of LC (**d**), ELC1 (**e**) and ELC2 (**f**); EDX spectra of LC (**g**), ELC1 (**h**) and ELC2 (**i**).

Figure 2(a) and (c) shows the digital photos of LC, ELC1 and ELC2. The fibers are disposed in a multidirectional array forming a natural mat-like structure. It is obviously see that the color of fibers becomes darker after modification. The SEM images of LC, ELC1 and ELC2 are shown in (d), (e) and (f). The surface morphology of LC appears smooth and neat. However, after grafting onto the matrix, the surface morphology of ELC1 and ELC2 turned out to be rough. As shown in EDX spectra of LC (g), ELC1 (h) and ELC2 (i), the atomic% of O decreases from 43.27 to 41.92 and 38.45 and N increases from 0.59 to 0.74 and 2.21 during route1 and route2.

As shown in Fig. S1, the FTIR spectra of LC (a), ELC1 (b) and ELC2 (c). Intense bands around 3400 and 1060 cm^−1^ are indicative of the existing hydroxyl groups in LC. In ELC1 the shift of the characteristic peak from 1632 to1643 cm^−1^ indicates the substitution of −COO^−^Na^+^ group on starch molecular chain during esterification of LC with EDTA^52^. The increasing intensity at 1740 cm^−1^ and the appearance of new peaks at 1261 cm^−1^ in ELC2 are attributed to the stretching vibration of C=O and C-N of -NHCO- derived from the reaction of amino and EDTA dianhydride.

The XRD pattern of LC, ELC1 and ELC2 were shown in Fig. S2. The characteristics peaks of LC were observed at 20.10° and 14.50° with relative intensities at 1341 and 808, respectively. The peaks of ELC1 and ELC2 were found at 20.12°, 14.48° and 20.24°, 14.46° with relative intensities of 933, 633 and 875, 625, respectively. The decrease of peak intensity indicates grafting to the backbone impairing the crystallinity of the fiber which might reduce hardness and increase flexibility.

Elemental analysis was used to further verify the preparation of ELC1 and ELC2 and calculated the graft ratios. Table 1 shows the results of LC, ELC1, epoxy-LC, amino-LC, and ELC2. The obvious increase of nitrogen content in ELC1 confirms the successful introduction of EDTA dianhydride onto LC; during the process of grafting EPI, the nitrogen content decreases and then increases for the reaction of epoxy rings with EDA. After introducing EDTA dianhydride, the nitrogen content continues to increase from 0.93 to 1.75%. The changes of nitrogen content indicate successful preparation of ELC2. By calculation the graft ratio of ELC1 and ELC2 are 15.01 and 21.44%, respectively. The results show that the new synthetic route effectively improves the graft ratio. The effect of graft ratio on adsorption of U(VI) is discussed latter.

### Adsorption analysis

The effect of pH on the adsorption capacity of U(VI) by ELC1 and ELC2 was evaluated in a pH range of 2.0–8.0. As shown in Fig. S3, it is clear that the adsorption capacity of both ELC1 and ELC2 toward U(VI) strongly depends on solution pH. The pH_zpc_ (pH zero point charge, Fig. S4) of ELC1 and ELC2 is found to be 2.0 and 2.1. When pH > 2.1 the surface charge of ELC1 and ELC2 is negative, which is prone to adsorb positively charged metal ions on their surfaces. However, at low pH abundant of hydronium ion (H_3_O^+^) competed with U(VI) for binding on the functional groups (binding sites). With the increase of pH, the adsorption capacity increases and reaches maximum at pH 6.0. When pH values are greater than 6.0, the prominent U(VI) species are multi-nuclear hydroxide and carbonate complexes such as (UO_2_)_2_(OH)_2_^2+^, (UO_2_)_3_(OH)^5+^ and (UO_2_)(CO_3_)_3_^4−^, leading to the decreased adsorption of U(VI)^53^,^54^. Therefore, an optimum pH value for effective adsorption was chosen as 6.0 for further studies.

**Figure 3:**
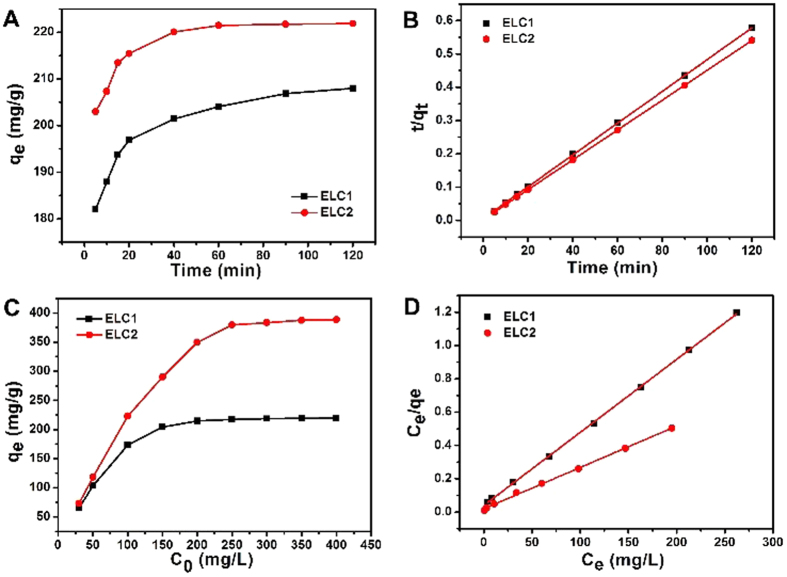
Effect of contact time of ELC1 and ELC2 (**A**) and their corresponding pseudo second-order kinetics (**B**); effect of initial concentration of ELC1 and ELC2 (**C**) and their corresponding Langmuir adsorption isotherms (**D**).

The effect of contact time was investigated in a kinetics study of the adsorption process. Figure 3A shows the time profile of U(VI) adsorption onto ELC1 and ELC2 in terms of adsorption capacity. It is observed that adsorption reaches an equilibrium in 60 min and 40 min (shorter than 120 min for LC) with an adsorption efficiency of 73.2% and 88.7% for ELC1 and ELC2, respectively. The results indicate that the new synthesis route not only increases the adsorption rate but also the adsorption efficiency.

The following pseudo-first-order^55^ (Fig. S5) and pseudo-second-order^56^ (Fig. 3B) models are employed to further interpret the kinetic data:


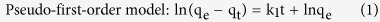



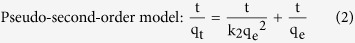


where q_e_ and q_t_ (mg/g) are the adsorption capacities at equilibrium and at time t (min), respectively; k_1_ and k_2_ are the rate constant of the pseudo-first-order and pseudo-second-order model.

The corresponding kinetic parameters from both models are listed in Table S1. Higher correlation coefficient (R^2^ > 0.99 for both) as well as value of q_e,cal_ (209.6 and 223.2 mg/g) of ELC1 and ELC2, approximating to q_e,exp_ (208.0 and 221.9 mg/g), indicate that a pseudo-second-order model describes the adsorption process better. This model is based on the assumption that the rate-limiting step of the reaction is due to chemical adsorption. Thus, more grafting EDTA is favorable for adsorption.

Figure 3C shows the equilibrium adsorption capacity of U(VI) onto ELC1 and ELC2 as a function of different initial uranium concentrations. The adsorption capacity increases rapidly with the initial concentration increasing until equilibrium is reached. The equilibrium adsorption capacities of ELC1 and ELC2 for U(VI) on are 219.25 and 388.25 mg/g.

The equilibrium adsorption isotherm is fundamental to describe the interactive behavior between the solution and adsorbent. The adsorption equilibrium data of U (VI) onto adsorbent is analyzed in terms of Langmuir and Freundlich models in this paper. The Langmuir isotherm assumes monolayer coverage on the surface of the adsorbent and no subsequent interaction among adsorbed molecules^57^. The linear Langmuir isotherm equation is given as:


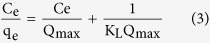


where Q_max_ (mg/g) is the maximum absorption capacity; K_L_ is Langmuir constant. The values of Q_max_ and K_L_ are calculated from the slope and intercept respectively from the linear plot of C_e_/q_e_ versus C_e_ (Fig. 3D).

Freundlich isotherm is an empirical equation which is used to describe adsorption at the multilayer and taking place on heterogeneous surfaces^58^. The equation of Freundlich isotherm is as follows:


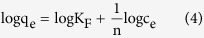


where q_e_ (mg/g) and C_e_ (mg/L) are the adsorption capacity and the equilibrium concentration of U(VI), respectively; K_F_ and n are Freundlich constants. The experimental data are plotted as log q_e_ versus log C_e_ (Fig. S6).

The model parameters obtained by Langmuir and Freundlich models are given in Table S2. The present adsorption process is likely dominated by a monolayer adsorption because the Langmuir isotherm fits a higher correlation coefficient for ELC1 and ELC2. This, as well as the calculated adsorption capacity (Q_max_, 228.3 and 416.7 mg/g), exhibit good agreement with the experimental values. As shown in Table S2, the grafting of EDTA effectively increases the adsorption capacity of LC; U(VI) coordinates with EDTA sodium to form a stable composite^59^. By comparison with ELC1, ELC2 had a bridge between LC and EDTA. But from the calculation of the ratio of Q_max,L_ and graft ratio compared with ELC1, adsorption of ELC2 is also mainly dependent on EDTA. The result indicates that the increase of the adsorption capacity is due to the increase of the graft ratio of EDTA.

Furthermore, the essential characteristics of the Langmuir isotherm are described by a separation factor, R_L_^60^, which is defined as follows:


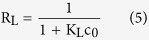


where K_L_ is the Langmuir equilibrium constant and C_0_ is the initial concentration of metal ion. The values of R_L_ give an indication for the possibility of the adsorption process to proceed: R_L_ > 1.0, unfavorable; R_L_ = 1, linear; 0 < R_L_ < 1, suitable; R_L_ = 0, irreversible^61^. The values of R_L_ lie between 0.1022 and 0.0084, indicating the suitability of ELC2 as adsorbent for U(VI) from aqueous solution.

To determine the effect of temperature on U(VI) adsorption, adsorption experiments were conducted at 298, 308 and 318 K. The equilibrium adsorption capacity of ELC1 and ELC2 increases with the increase of temperature (from 219.2 and 388.2 to 254.5 and 458.7 mg/g), indicating that the adsorption of U(VI) onto EDTA modified LC is an endothermic process.

The thermodynamic parameters, enthalpy (ΔH°) and entropy (ΔS°), associated with the adsorption process were calculated from the Van’t Hoff equation:


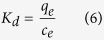



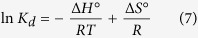


where Δ*H*° (kJ mol^−1^) and Δ*S*° (J mol^−1^ K^−1^) are enthalpy and entropy, respectively; *R* (8.314 J mol^−1^ K^−1^) is the universal gas constant; *T* is the absolute temperature (K); *q*_e_ (mg g^−1^) and *C*_e_ (mg L^−1^) are the adsorption capacity and the concentration of U(VI) at equilibrium, respectively. The plot of ln *Kd* as a function of 1/*T* yields a straight line from which Δ*H*° and Δ*S*° are calculated from the slope and intercept, respectively. The thermodynamic parameters calculated are tabulated in Table S3.

The positive value of Δ*H*° shows the endothermic nature of adsorption process, coinciding with the practical result. A higher temperature favors adsorption. The Gibbs free energy of specific adsorption (Δ*G*°) was calculated using the equation:


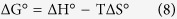


The negative values of Δ*G*° indicate that the adsorption reaction of EDTA modified LC is spontaneous.

**Figure 4:**
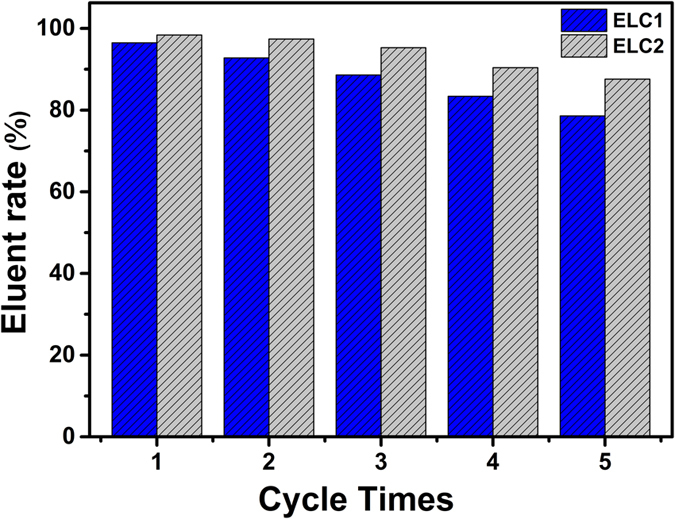
Regeneration studies of ELC1 and ELC2.

According to the previous result, ELC1 and ELC2 have poor U(VI) adsorption ability under acid conditions. HNO_3_ (0.1–1.0 mol/L) was used to evaluate the reutilization performance of ELC1 and ELC2. The results obtained are given in Table S4. The elution efficiency is above 95% when the acid concentration is greater than 0.6 mol/L, suggesting that HNO3 is an effective eluent for the recovery of the adsorbent. Therefore, reusability experiments were subsequently performed using 0.6 mol/L HNO_3_ (Fig. 4). It is observed that regenerated ELC2 still had high adsorption capacity even after 5 cycles of adsorption/desorption, indicating that ELC2 is a stable and recyclable adsorbent. The decrease in adsorption capacity of ELC1 is probably due to hydrolysis of the ester bond between EDTA and LC fiber as well as ELC1 disintegration at high acidity.

According to the above, ELC2 has better adsorption properties than ELC1, including shorter equilibrium time, higher adsorption capacity and better reutilization performance. The results show that the new synthetic route of grafting EDTA onto LC fiber successfully improves not only the ratio of grafting, but also the adsorption property.

### Uranium adsorption tests in simulated seawater

**Figure 5:**
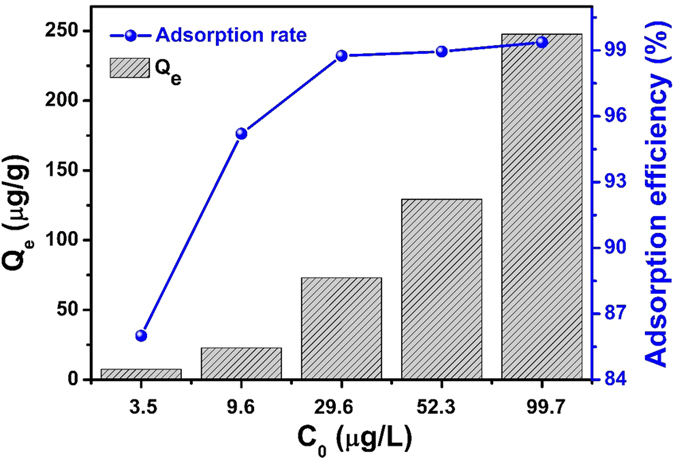
The adsorption rate of U (VI) by ELC2 in simulated seawater.

To further investigate whether ELC2 is suitable for the extraction of uranium from seawater, we examined the applicability of adsorbent in the extraction of low concentration U(VI) from aqueous (U(VI) only) and simulated seawater (U(VI) and other metals ions with similar or higher concentrations). As shown in Table S5, ELC2 shows excellent adsorptive capacity from aqueous, even at extremely low concentration of 3 μg/L (residual concentration <0.1 μg/L). In simulated seawater, U(VI) is still effectively adsorbed onto ELC2 and the adsorption rate is above 85% (Fig. 5). The results show that ELC2 can be able to extract U(VI) in the presence of other metals ions. It is interesting to note that residual U(VI) in simulated seawater is almost the same, at an initial concentration of 100 μg/L as well as 3 μg/L (Table S6). The possible reason for this is that the adsorption of U(VI) on ELC2 in solution reaches an equilibrium from which little further progress is made.

**Figure 6:**
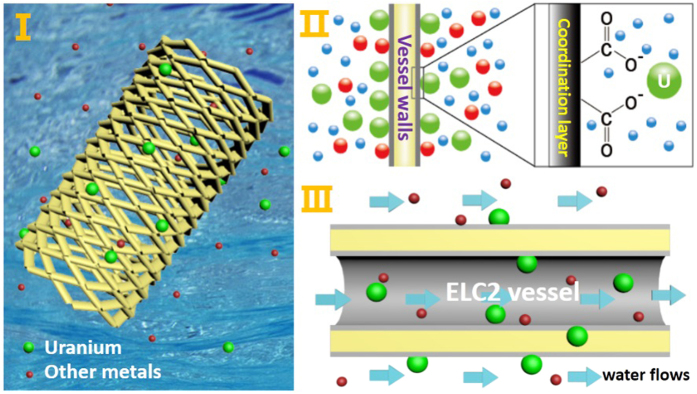
U(VI) adsorption on ELC2 in simulated seawater (**I**); U(VI) adsorb on the coordination layer of vessel walls (**II**); water flows from vessel (**III**).

According to previous studies, it is reasonable to attribute such high adsorption ability of ELC2 for U(VI) to two factors (Fig. 6). On the one hand, the new approach of grafting EDTA onto LC generates a greater graft ratio and EDTA exhibits strong coordination to U(VI) (Fig. 6II). On the other hand, L. cylindrica has a highly porous fibrous vascular system in which the fibers are disposed in a multidirectional array forming a natural mat-like structure (Fig. S7 and Table S7). The unique hydrophilic structure makes seawater quickly flow from the vascular and promoted the coordination to U(VI) (Fig. 6III).

### Conclusions

In summary, we demonstrate a better strategy for introducing EDTA onto L. cylindrica than one-step esterification. The new route in this paper effectively improved not only the graft ratio (5.48 to 10.46%), but also adsorption properties, such as shorter equilibrium time (60 min to 40 min), higher adsorption capacity (219.25 to 388.25 mg/g) and better reutilization performance (fifth cycle 78.6% to 87.6%). Moreover, combining the merits of a unique porous fibrous vascular structure of L. cylindrica, the adsorbent, as prepared by this new synthetic route, makes efficient contact with a low concentration of U(VI) in simulated seawater. We conclude that EDTA modified L. cylindrica will be potentially advantageous in the extraction of uranium from seawater.

## Methods

### Materials

L. cylindrica was purchased from Handan, China. Ethylenediamine tetraacetic acid (EDTA) was purchased from Xiya (China). Other organic reagents, including EPI and EDA were purchased from Fuyu (China). NaOH, HCl and NaHCO_3_ were supplied by Zhiyuan (China). All reagents were of AR grade and were used without further purification.

## Preparation of adsorbent

### Pretreatment of L. cylindrica

L. cylindrica fibers were obtained from a dried fruit and the outer mat was utilized in this study. Firstly, L. cylindrica was immersed in fresh water at room temperature for about 4 days to remove dirt and soluble impurities. Then it was treated with 10% sodium hydroxide for 60 min to increase its hydrophilicity. The alkali treated fibers were washed thoroughly with distilled water until the pH was close to neutral. The fibers were finally dried in an oven at 328 K for 12 h and denoted LC.

### Preparation of EDTA-LC

#### Route 1

EDTA was first made into the form of acid anhydride following the methodology described by Capretta *et al*.^62^. Then the prepared EDTA dianhydride (1.0 g) and LC (1.0 g) were added to N,N-dimethylformamide (DMF) (80 mL) in a flask. After the mixture was stirred at 338 K for 4 h, the product was washed with DMF, saturated sodium bicarbonate solution and deionized water, successively. The EDTA modified LC fibers thus obtained were dried at 328 K for 12 h in an oven and denoted as ELC1.

#### Route 2

##### Preparation of epoxy-LC

LC (1.0 g) was immersed in NaOH solution (50 ml, 5%), EPI (15 ml) and alcohol (15 ml) to introduce epoxy groups onto the on the surface of fibers. The mixture was left under stirring at 328 K for 5 h. The treated fibers were washed with distilled water to a neutral pH and then dried in an oven at 328 K^63^,^64^.

##### Preparation of amino-LC

The amino groups were anchored onto the fiber by aminolyzation via a ring-opening reaction between the epoxy group and EDA. Briefly, epoxy-LC (1 g), EDA (5 ml) and Na_2_CO_3_ solution (50 ml, 1%) were added to a round-bottom flask and stirred at 328 K for 2 h. Then the product was filtered, rinsed thoroughly with water and dried at 328 K^65^,^66^.

##### Preparation of EDTA-LC

After EDA modification, amino-LC (1 g) and EDTA dianhydride (1.0 g) were added to DMF (80 mL) (the same amount of reactants as Route 1). The mixture was stirred at 338 K for 2 h. The product was filtered and washed with DMF, saturated sodium bicarbonate solution and deionized water, successively. The final EDTA modified LC was dried in an oven for 12 h at 328 K and denoted as ELC2.

## Batch Equilibrium U(VI) Adsorption Experiments

Individual stock solution of 1000 mg/L U(VI) was prepared by dissolving UO_2_(NO_3_)_2_·6H_2_O in deionized water. Adsorption experiments were performed in a 50 mL U(VI) solution with initial concentrations of 30, 50, 100, 200, 250, 300, 350, or 400 mg/L, 20 mg adsorbent and the solution pH adjusted to 6 with HNO_3_ or NaOH. The effect of solution pH was evaluated in the case of 100 mg/L U(VI), 20 mg adsorbent and where the contact time was 120 min. For kinetics studies, the solution was shaken at 200 rpm for 5, 10, 15, 20, 40, 60, 90 or 120 min. After adsorption, the adsorbent was collected and regenerated by using 50 mL HNO_3_. An enhanced simulated seawater test was performed following our previous work^23^. ELC2 was added into 50 mL simulated seawater containing U(VI) with initial concentrations of 3, 10, 30, 50 or 100 μg/L, after shaking at 200 rpm for 120 min, the residual U(VI) ions in solution were analysed by ICP-MS.

### Characterization methods

Qualitative chemical structure assessment was done by FT-IR analysis (PerkinElmer Spectrum 100) in a range of 4000–500 cm^−1^. The element content was measured by element analyzer (Vario Macro). Inductively coupled plasma-atomic emission spectroscope (ICP-AES, Optima-7000DV) was used to analyze the initial and equilibrium concentration of U(VI). The concentration of trace U(VI) was analyzed using inductively coupled plasma mass spectrometry (ICP-MS, Bruker 820-MS).

## Additional Information

**How to cite this article**: Su, S. *et al*. Enhancing adsorption of U(VI) onto EDTA modified L. cylindrica using epichlorohydrin and ethylenediamine as a bridge. *Sci. Rep.* **7**, 44156; doi: 10.1038/srep44156 (2017).

**Publisher's note:** Springer Nature remains neutral with regard to jurisdictional claims in published maps and institutional affiliations.

## Supplementary Material

Supplementary Information

## Figures and Tables

**Table 1:** Elemental analysis results of raw-LC, LC, ELC1, epoxy-LC, amino-LC, and ELC2.

Compound	Elemental content %		
	C	N	H
LC	43.81	0.23	6.14
ELC1	42.43	0.66	6.03
epoxy-LC	44.13	0.19	6.12
amino-LC	43.97	0.93	6.33
ELC2	42.24	1.75	6.00
