# Supplementary material for: Enhancing adsorption of U(VI) onto EDTA modified L. cylindrica using epichlorohydrin and ethylenediamine as a bridge
Source: Sci Rep. 2017 Mar 8;7:44156. doi: 10.1038/srep44156 (PMC5341152; doi:10.1038/srep44156)
Supplement: Supplementary Information [file srep44156-s1.doc]

*Electronic Supplementary Information for*

Enhancing adsorption of U(VI) onto EDTA modified L. cylindrica using epichlorohydrin and ethylenediamine as a bridge

Shouzheng Su,^a^ Qi Liu,^bc^* Jingyuan Liu,^a^ Hongsen Zhang,^d^ Rumin Li,^a^ Xiaoyan Jing,^a^ Jun Wang ^abc^*

a. Key Laboratory of Superlight Material and Surface Technology, Ministry of Education, Harbin Engineering University, 150001, P. R. China.

E-mail:zhqw1888@sohu.com:Fax:+86 451 8253 3062:Tel: +86 451 8253 3062

b. Institute of Advanced Marine Materials, Harbin Engineering University, 150001, P. R. China.

c. Harbin Shipbuilding Engineering Design & Research Academy, Harbin, China.

d. Modern Analysis, Test and Research Center, Heilongjiang University of Science and Technology, Harbin 150027, PR China.

Tab. S1. Kinetic parameter for adsorption of U(VI) on ELC1 and ELC2

| Kinetics model | Materials | K | q_e,exp_(mg/g) | q_e,cal_(mg/g) | R^2^ |
| --- | --- | --- | --- | --- | --- |
| Pseudo-first  order | LC | 4.273*10^-2^ | 76.8 | 69.82 | 0.9524 |
|  | ELC1 | 5.179*10^-2^ | 208.0 | 40.89 | 0.8883 |
|  | ELC2 | 6.124*10^-2^ | 221.9 | 22.62 | 0.9817 |
| Pseudo-second order | LC | 9.340*10^-4^ | 76.8 | 80.31 | 0.9964 |
|  | ELC1 | 3.889*10^-3^ | 208.0 | 209.64 | 0.9994 |
|  | ELC2 | 7.117*10^-3^ | 221.9 | 223.21 | 0.9999 |

Tab. S2. Isotherm parameter for adsorption of U (VI) on LC, ELC1 and ELC2

| Materials | Langmuir isotherm | | | Freundlich isotherm | | |
| --- | --- | --- | --- | --- | --- | --- |
|  | Q_max_ (mg/g) | K_L_(L/mg) | R^2^ | K_F_(L/mg) | n | R^2^ |
| LC | 75.47 | 0.6548 | 0.9990 | 20.70 | 4.319 | 0.8232 |
| ELC1 | 228.3 | 0.1090 | 0.9981 | 54.38 | 3.608 | 0.8834 |
| ELC2 | 416.7 | 0.1276 | 0.9990 | 1.572 | 3.414 | 0.9484 |

Tab. S3. Thermodynamic parameters of uranium adsorption on ELC1 and ELC2

| Materials | △H° (kJ mol^-1^) | △S°(J mol^-1^ K^-1^) | △G° (kJ mol^-1^) | | |
| --- | --- | --- | --- | --- | --- |
|  |  |  | 298K | 308K | 318K |
| ELC1 | 5.79 | 34.45 | -4.46 | -4.80 | -5.14 |
| ELC2 | 7.34 | 43.60 | -5.56 | -6.08 | -6.52 |

Tab. S4. The elution efficiency upon the different concentration of HNO_3_.

| HNO_3_ concentration  (mol/L) | Elution efficiency (%) | | |
| --- | --- | --- | --- |
|  | LC | ELC1 | ELC2 |
| 0.2 | 83.2 | 73.6 | 84.3 |
| 0.4 | 87.6 | 84.8 | 90.2 |
| 0.6 | 95.7 | 96.5 | 98.4 |
| 0.8 | 96.4 | 98.6 | 99.3 |
| 1.0 | 98.8 | 99.7 | 99.5 |

Tab. S5. The extraction of U(VI) by ELC2 in aqueous.

| Initial concentration (μg/L) | 3.2 | 10.3 | 31.2 | 51.4 | 100.9 |
| --- | --- | --- | --- | --- | --- |
| Residual concentration (μg/L) | 0.08 | 0.11 | 0.14 | 0.17 | 0.15 |
| Adsorption rate (%) | 97.5 | 98.9 | 99.6 | 99.8 | 99.4 |

Tab. S6. The extraction of U(VI) by ELC2 in simulated seawater.

| Initial concentration (μg/L) | 3.5 | 9.6 | 29.6 | 52.3 | 99.7 |
| --- | --- | --- | --- | --- | --- |
| Residual concentration (μg/L) | 0.49 | 0.46 | 0.37 | 0.55 | 0.63 |
| Adsorption rate (%) | 86.0 | 95.2 | 98.8 | 98.9 | 99.4 |

Tab. S7. The pore volume and BET of ELC2.

| Materials | Pore volume (mL/g) | Median pore diameter (nm) | Porosity (%) | BET (m^2^/g) |
| --- | --- | --- | --- | --- |
| ELC2 | 1.4325 | 1989.1 | 55.6 | 230 |


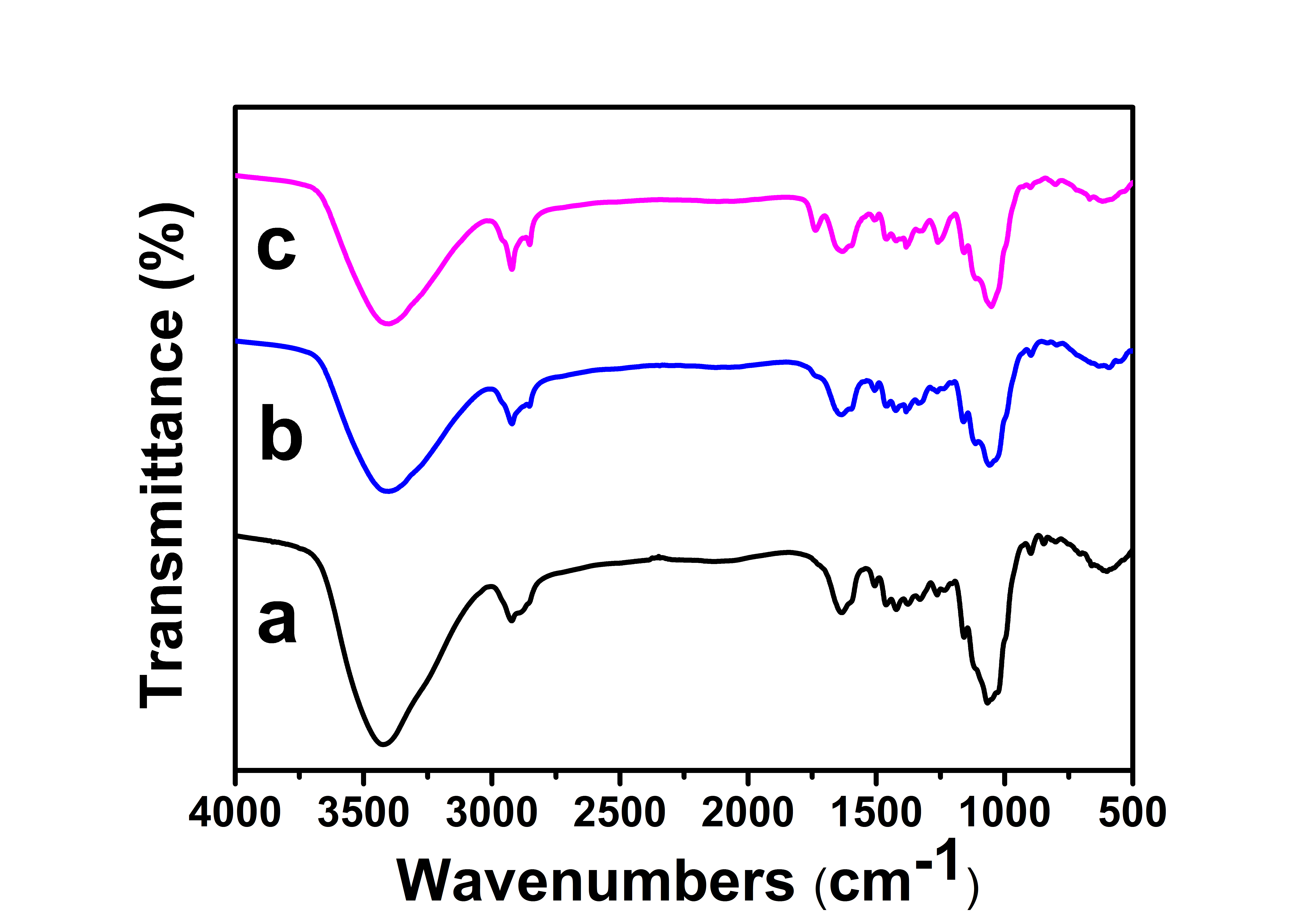


Fig. S1. FTIR spectroscopy of LC, ELC1 and ELC2.


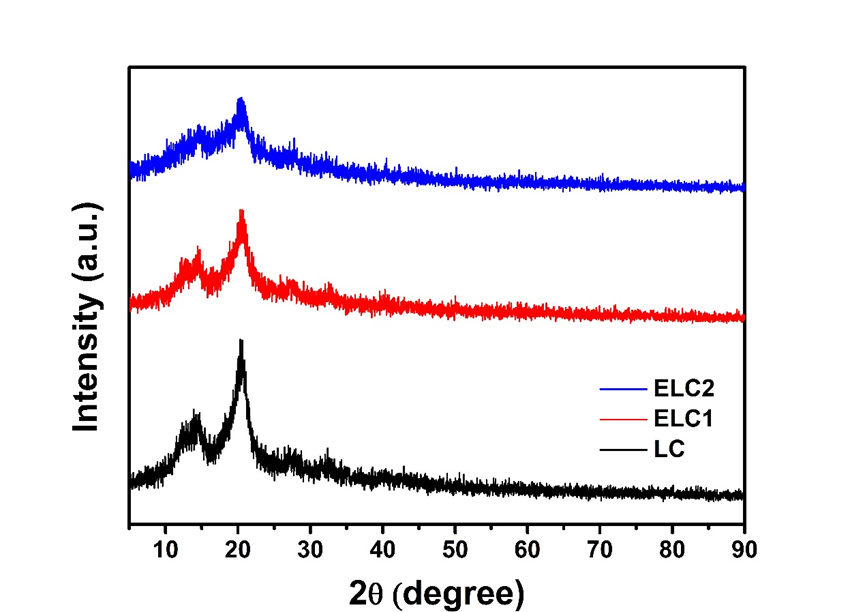


Fig. S2. XRD spectra of LC, ELC1 and ELC2.


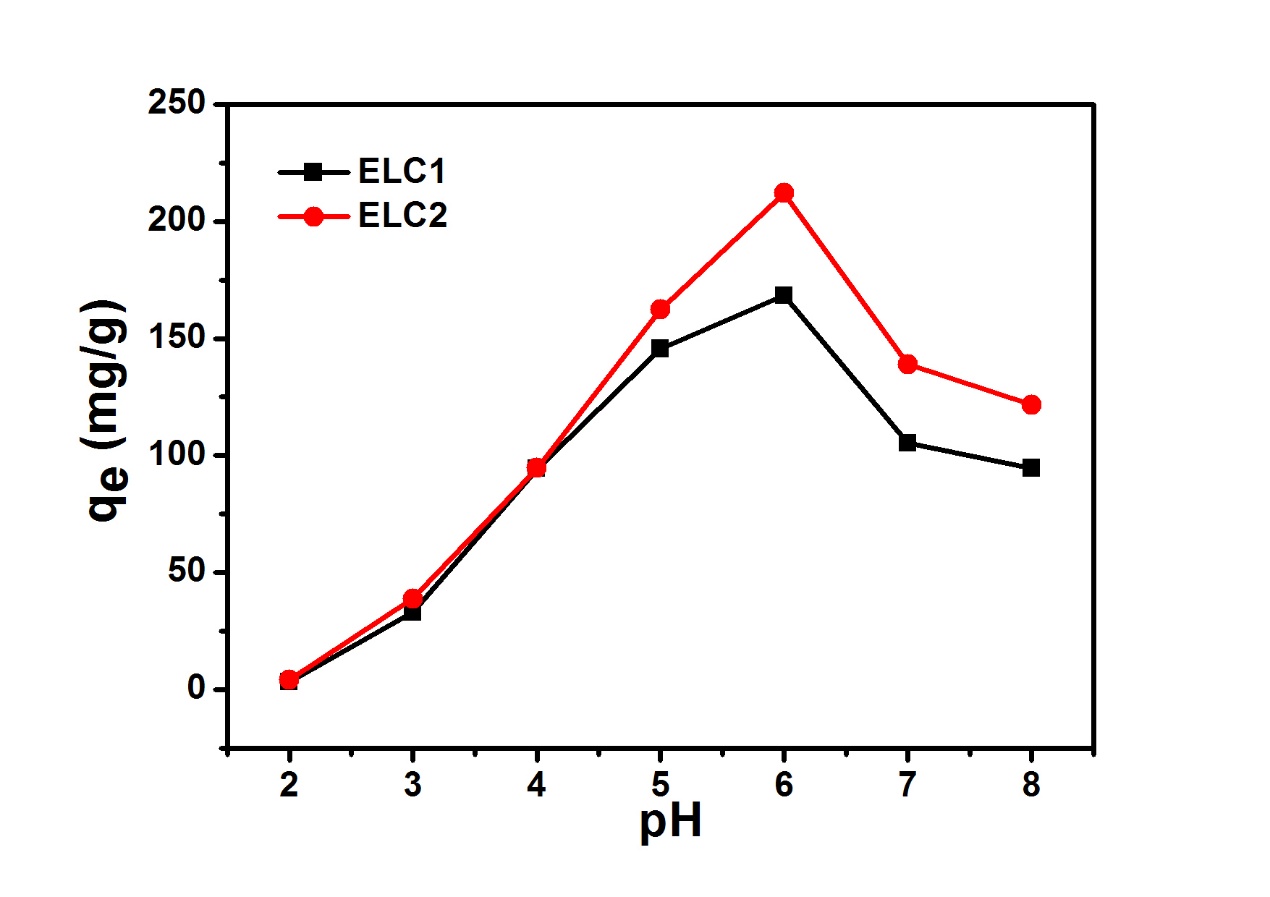


Fig.S3. Effect of pH on adsorption capacity of ELC1 and ELC2.


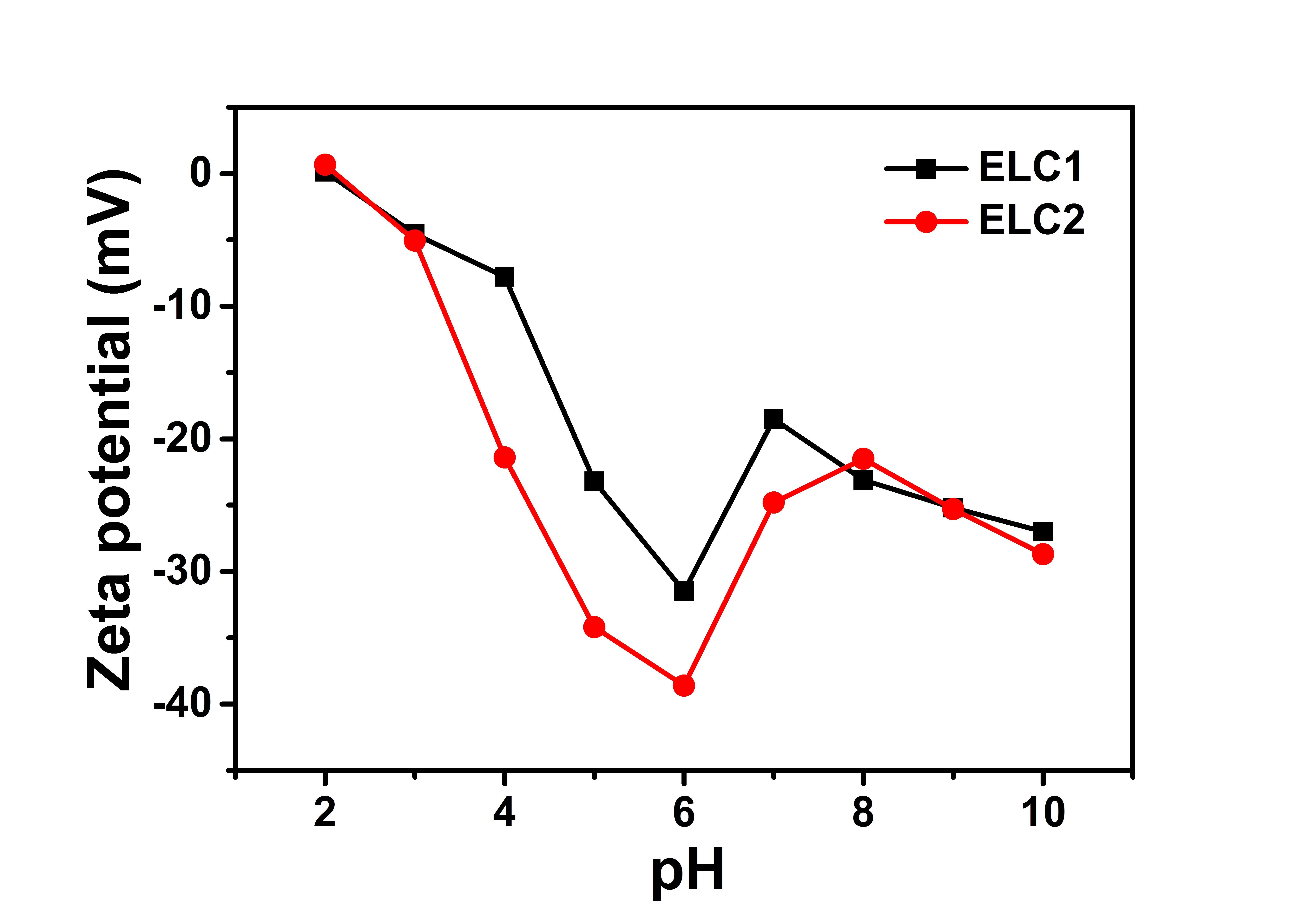


Fig. S4. Zeta potential of ELC1 and ELC2.


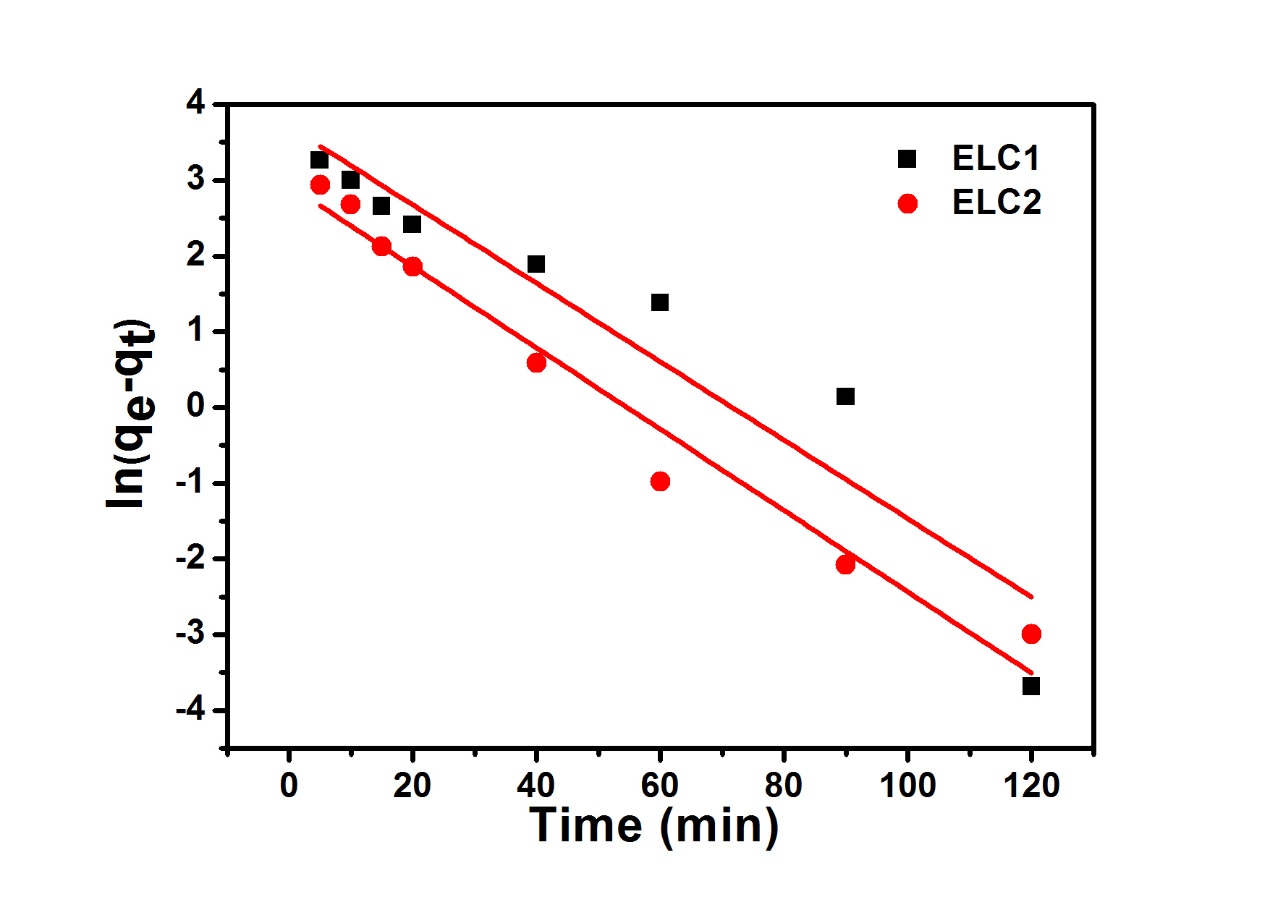


Fig. S5. Pseudo-first-order linearly fitted curve.


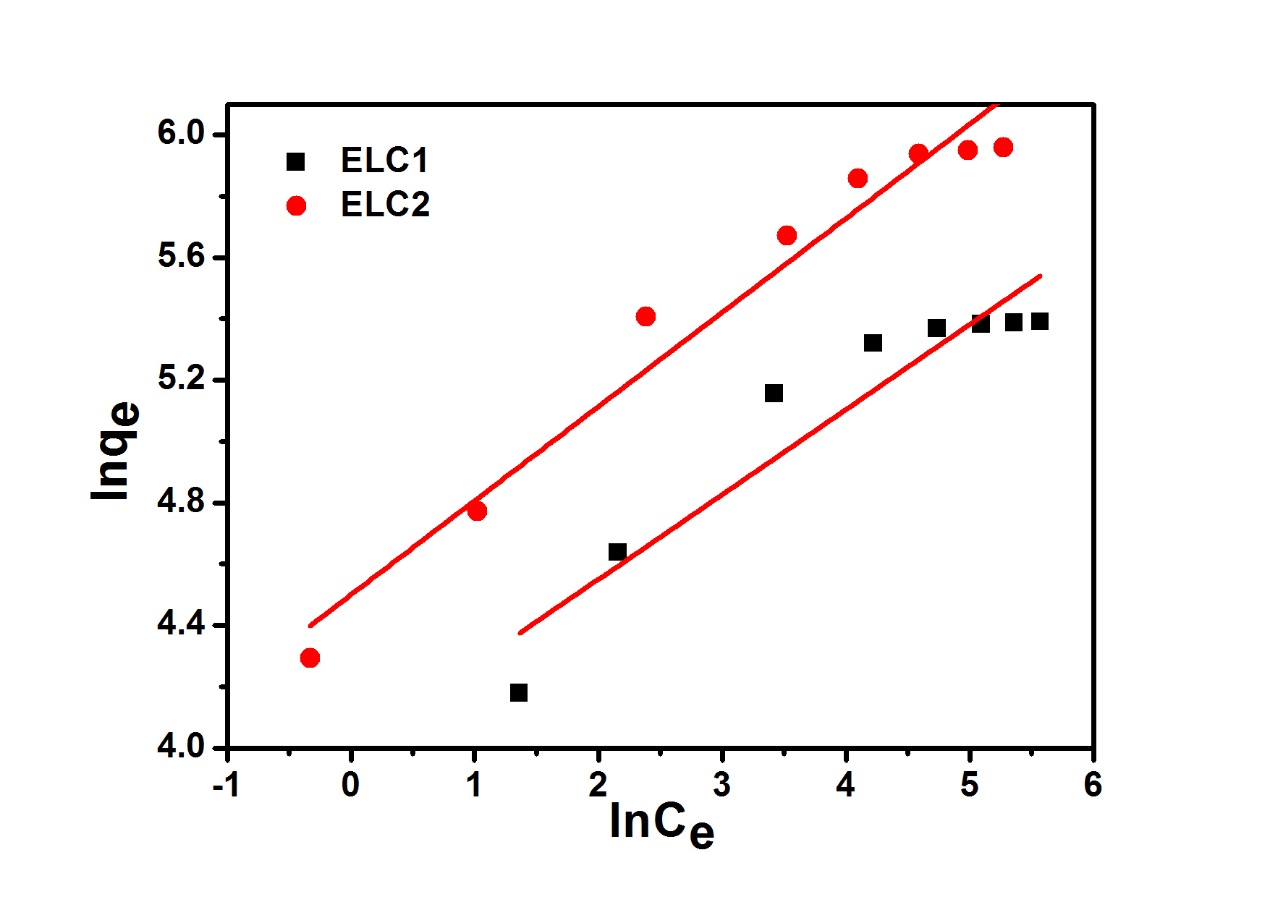


Fig. S6. Freundlich isotherm.


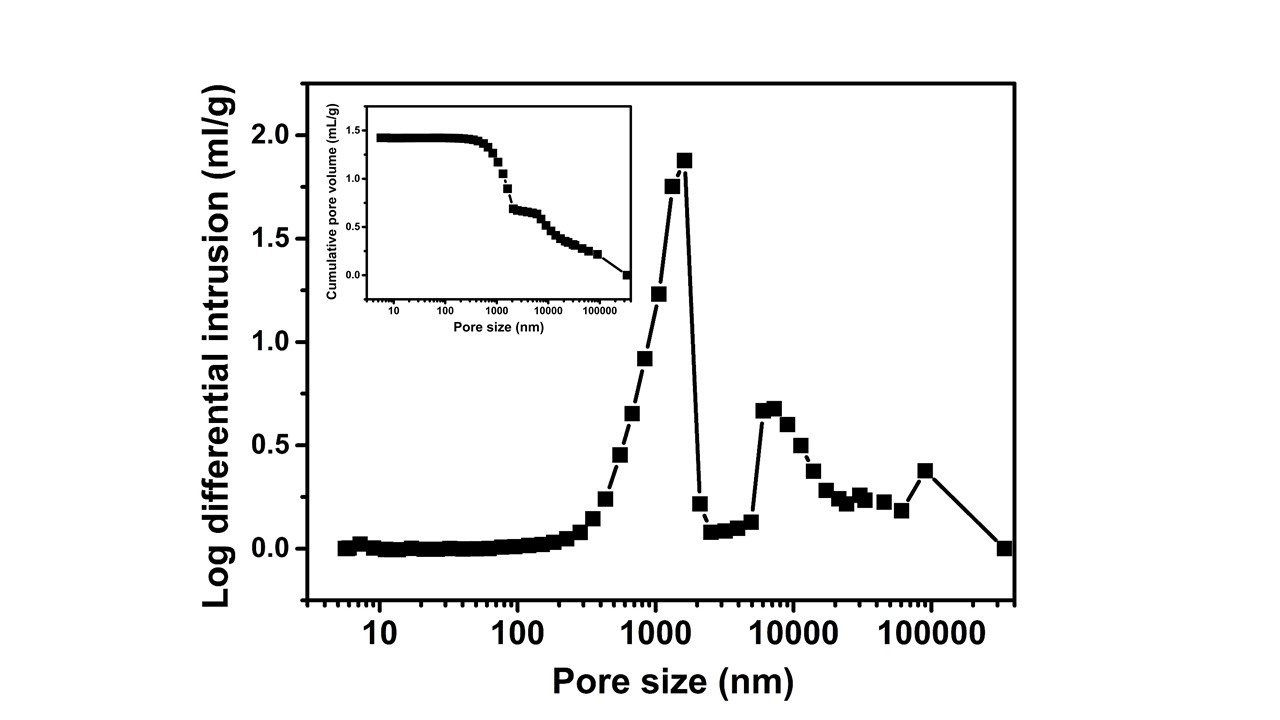


Fig. S7. Pore size distribution of ELC2.
